# Firm level optimisation strategies for sustainable and cost effective electric vehicle workplace charging

**DOI:** 10.1038/s44333-025-00032-w

**Published:** 2025-03-17

**Authors:** Marcel Seger, Christian Brand, Christoph Clement, James Dixon, Charlie Wilson

**Affiliations:** 1 Environmental Change Institute, University of Oxford, Oxford, UK; 2 Transport Studies Unit, University of Oxford, Oxford, UK; 3 Center for Artificial Intelligence in Medicine, University of Bern, Bern, Switzerland; 4 Department of Civil and Environmental Engineering, University of Strathclyde, Glasgow, UK; 5 International Institute for Applied Systems Analysis, Laxenburg, Austria

**Keywords:** Operational research, Industry, Energy management

## Abstract

Expanding electric vehicle (EV) charging infrastructure is essential for transitioning to an electrified mobility system. With rising EV adoption rates, firms face increasing regulatory pressure to build up workplace charging facilities for their employees. However, the impact of EV charging loads on businesses’ specific electricity consumption profiles remains largely unknown. Our study addresses this challenge by presenting a mathematical optimisation model, available via an open-source web application, that empowers business executives to manage energy consumption effectively, enabling them to assess peak loads, charging costs and carbon emissions specific to their power profiles and employee needs. Using real-world data from a global car manufacturer in South East England, UK, we demonstrate that smart charging strategies can reduce peak loads by 28% and decrease charging costs and emissions by 9% compared to convenience charging. Our methodology is widely applicable across industries and geographies, offering data-driven insights for planning EV workplace charging infrastructure.

## Introduction

Efforts to mitigate climate emissions in the transport sector strongly focus on road vehicle electrification: 65% of commitments in nations’ revised nationally determined contributions as of 2021 centre on electrification and fuel-switching^1^. For road vehicle electrification to continue at pace in delivering these commitments, widespread charging infrastructure at workplaces and public places is essential to achieve ‘convenience parity’ between electric vehicles (EVs), referring to private passenger cars solely powered by an electric battery and motor, and internal combustion vehicles^2^. Given the significance of workplaces as car parking locations, EV charging at workplaces plays a crucial role in facilitating the rapid electrification of private passenger transport. For instance, an estimated 21% of car-based trips and 25% of car-based kilometres were taken for commuting purposes in the UK in 2022^3^. Furthermore, the proliferation of workplace charging is expected to distribute the load of EV charging in space and time, thereby reducing the evening peak of—and thus stress placed on—electricity distribution networks^4^.

For workplace charging to contribute to the future landscape of energy provision for EVs, workplaces must be incentivised to provide it. There are two plausible benefits to workplaces installing chargers: providing charging can help attract and retain workers^5^, and workplaces can aggregate EV batteries to provide services to the electricity grid, representing an extra stream of revenue^6^. Such grid services, including frequency response^7^ and peak-shaving^8^, all ultimately result from the shifting (in time) and the modulation of power flows to and from the EV battery^9^. The general concept of optimising EV charging processes towards one or multiple objectives, such as minimising charging costs or carbon emissions while ensuring users’ state-of-charge (Soc) preferences are met, is referred to as ‘smart charging’ (SC)^10^.

More broadly, from a system-level electricity network perspective, an extensive body of research has analysed the synergetic effects of utilising SC (V1G) and vehicle-to-grid (V2G) mechanisms as key enabler for active control in distribution grids. To this end, Kempton and Tomić ^11^ computed quantitative estimates of the relative fleet size of distributed V2G-capable EVs, based on the US electricity system, to provide enhanced grid-stability services. Similarly, Lund and Kempton^12^ modelled the demand-side flexibility potential arising from V2G-enabled EVs to achieve higher penetration rates of renewables while reducing total carbon emissions. In practical terms, to realise the full potential of V2G in active distribution grids, advanced coordination mechanisms between EVs and grid operators are required for participation in flexibility markets ^13^, typically managed by EV aggregators^14,15^.

Apart from modern software to manage EV charging cycles, smart infrastructure, including Internet-of-Things-enabled charge points, is indispensable as the backbone of a more flexible, intelligent network, commonly referred to as ‘smart grid’^16^. Lopes et al.^17^ developed an extensive framework showing the relation between technical management and market operation for effective EV integration into power systems, drawing out the hierarchical structure of different control levels between SC points and EVs. More recently, Shang et al.^18,19^ have modelled the interplay between the operation of decentralised SC points and the decision for optimal energy dispatching as cyber-physical system in the context of V2G scheduling. By contrasting different network typologies with varying numbers of nodes representing individual SC points, it delivers technical details on the engineering implementation of smart charge point infrastructure.

In the workplace context, Zheng et al.^20^ found that workplace EV charging has significant potential to provide grid services, benefiting both the network operator and the EV owner. While there has been growing research interest in the benefits of providing grid services from EV batteries in recent years^21–28^, all the reviewed works in the literature have focused on the level (or existence) of benefits to network operators or electricity market agents^21–24^ and EV owners^25–28^. Generally speaking, though, the decision to develop workplace charging infrastructure typically lies with the workplace itself. There is a need for workplaces to understand the potential benefits of installing and operating EV workplace charging infrastructure, including the trade-offs between different charging strategies and their associated impact on economic and environmental sustainability. However, there is a notable research gap in the provision of methods, data and tools to enable this.

To address this gap, we present a decision support tool for workplaces, allowing them to visualise the potential benefits and trade-offs from installing and operating EV charging infrastructure in terms of (i) reduced electricity costs, (ii) the provision of local grid services through peak minimisation and valley filling, and (iii) the reduction in carbon emissions resultant from the vehicles’ charging. All of these benefits have direct or indirect monetary rewards, which can be evaluated against the required investments in building business cases for charging infrastructure installation, ultimately supporting the growth in EV charging infrastructure and thus the viability of a rapid transition.

Our work contributes to the scientific discourse of EV workplace charging in three ways: First, in terms of methodological advancement, we utilise the core mathematical optimisation model by Ioakimidis et al.^29^ and expand its analytical scope to include economic- and environmental sustainability-related objective functions by drawing on a synthesis of modelling approaches compiled by Zheng et al.^30^. Second, we apply the augmented method in the context of a large-scale industrial manufacturing site and compute quantitative estimates of key output metrics based on real-world electricity consumption data. Third, fostering reproducibility and replicability of our results and encouraging wider application of our method and the generalisable insights beyond the case study presented, we make the optimisation algorithms publicly accessible by turning them into an open-source web app for academics and practitioners alike.

The novel contribution of this paper lies in the applicability of a linear optimisation model for supporting complex decision trade-offs specifically for workplace charging within a large industrial firm, utilising real-world electricity consumption data and considering unique firm-specific factors, such as shift patterns, and number of employee cars. The model integrates linear optimisation with the goal of minimising peak electricity demand, costs or emissions into a real-world, industry-specific context to inform workplace decision making on EV charging infrastructure. Furthermore, the open-source web application developed for this study empowers business executives with a customisable, user-friendly tool that can be applied directly to their sites, offering a practical solution that extends the utility of existing optimisation models. This real-world application, coupled with the transparency of the open-source web application, presents a contribution to an identified gap in the literature.

The rest of this paper is organised as follows: First, we briefly introduce the core case study-related information, before we present the main model results obtained from the scenario analysis concerning varying EV adoption rates. Subsequently, we assess the robustness of these model results by conducting a temporal sensitivity analysis, i.e. computing 28 single-day model runs for February 2023. Furthermore, we discuss the wider applicability of this work beyond the case study presented and discuss limitations and opportunities for further research. Lastly, we provide a detailed summary of the methods used.

## Results

### Core case study information

This case study examines a global car manufacturing firm with a production site in South East England, UK, that exhibits steep increases in electricity demand during plant operation. Given the high dependency on manual labour for assembly-related tasks, employees’ arrival by car at the plant before each shift coincides with the production ramp-up. If EVs were to be charged in an uncontrolled (UCC) manner, this would result in an additional peak, potentially surpassing the site’s power capacity limit. Instead, SC shifts the load across the entirety of the work shift, thereby decreasing the additional peak incurred from EV charging.

**Fig. 1:**
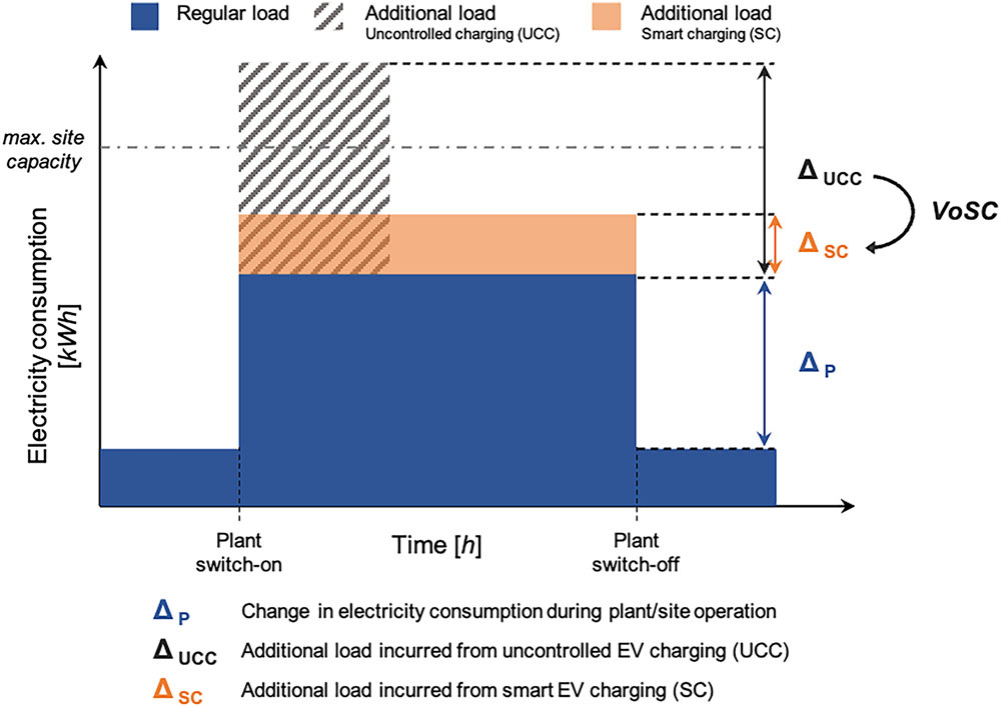
Schematic electricity consumption profile of industrial site. Note that this figure is for illustrative purposes only, visually explaining the concept of load shifting. In reality, charging loads of EVs, similar to the electricity consumption profile of the plant, play out in irregular shapes.

Figure 1 provides a schematic overview of the site’s electricity demand profile. For this study, we quantify the ‘*value of smart charging (VoSC)*’ by introducing the ‘*output change metric*’ that measures the relative change [%∆] between SC and UCC for each key metric (*ω*), where *ω* captures the resulting output in terms of maximum peak, total charging costs, or carbon emissions. It is computed according to eq. (1):

1 $$VoSC\ [ \% \Delta ]=\frac{\left({\omega }^{SC}-{\omega }^{UCC}\right)}{{\omega }^{UCC}}* 100$$

The manufacturing environment operates on a two-gear shift pattern for employees working in assembly, in addition to a single shift for office staff. The morning shift (AM) operates between 06:15–16:00, followed by the evening shift (PM) that runs between 16:15–02:00, with no production taking place between 02:00–06:15. Working hours of office staff range from 08:00–16:00. Supplementary Fig. 5a provides a visual overview of the actual electricity consumption profile of the industrial site, overlaid with the work shift schedules. We estimated 1100 unique employee cars to be coming to the workplace each day which we derived from real-world observations of parked cars on-site using video footage (CCTV, drone imagery), provided by our case study partner, at certain points throughout the day which are indicative of the car parks’ occupancy levels during each shift. Further details on the general modelling approach, in addition to case study-specific assumptions and input parameters, are presented in the ‘Methods’ section.

### Scenario analysis of EV adoption rate [%]

The initial analysis assesses the impact of increasing EV adoption rates on the total electricity consumption profile of the industrial site. We selected February 1st, 2023 as the reference date of analysis, as it exhibits a representative consumption profile of a typical working day (cf. Supplementary Fig. 5a), with a dip in demand during non-production hours (02:00–06:15) when no employee cars are parked, followed by a steep increase in demand as a result of production ramp-up.

We present the results from a prospective ‘what-if’ scenario analysis with increasing EV adoption rates as an exogenous input parameter, ranging from 15 to 100% [Scenarios S1–S5: 15, 30, 50, 80, 100%], by quantifying the VoSC for three different SC objectives (model types): Peak minimisation & valley filling (PM-VF), charging cost- (CCM), and carbon emission minimisation (CEM). To compute the VoSC (eq. (1)), each model output is benchmarked against UCC with respect to the key output metrics maximum peak, total charging costs, and carbon emissions.

**Fig. 2:**
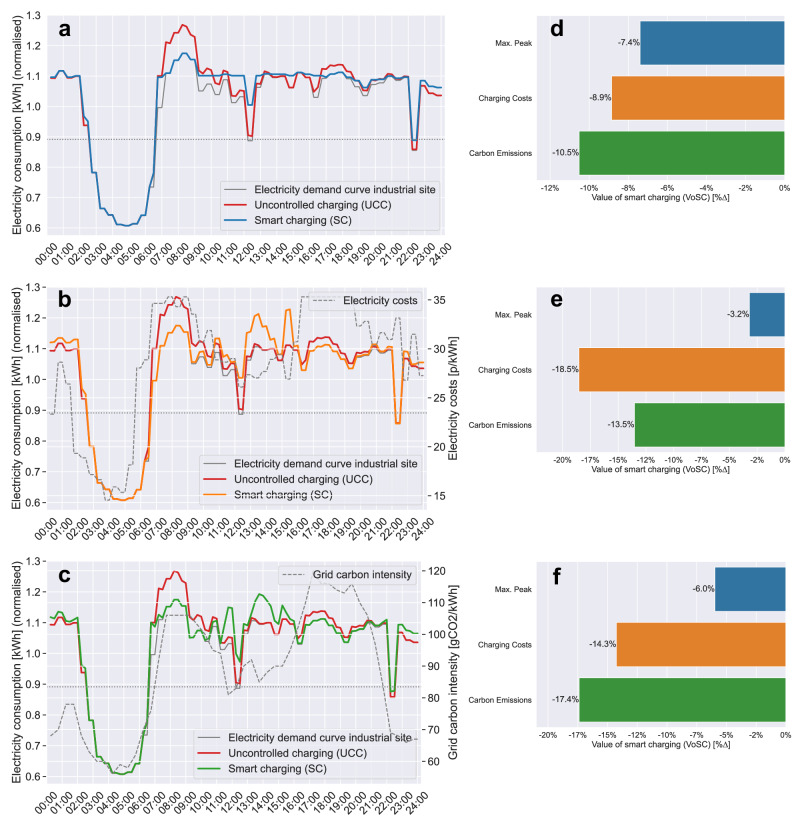
Charging profiles differentiated by model type for scenario 1 [S1: EV rate=15%]. Time-series analysis of daily charging loads for each objective function. Visualising the impact of EV workplace charging for (**a**) PM-VF, (**b**) CCM and (**c**) CEM by contrasting the resulting total electricity demand curve (*P*_*t*_ + *y*_*t*_) with the site’s electricity consumption profile *P*_*t*_ only and the total charging load incurred from UCC (*P*_*t*_ + *UCC*), in addition to plotting the exogenous price (*λ*_*t*_) [p/kWh] and grid carbon intensity (*γ*_*t*_) [gCO_2_/kWh] parameters. Quantifying the ‘Value of smart charging (VoSC)’ (relative change in output [%∆]) for each model type (**d**) PM-VF, (**e**) CCM and (**f**) CEM, measured against UCC, for the three key metrics max. peak demand [kWh], total charging costs [£] and total carbon emissions from charging [kgCO_2_]. Note that the y-axis displays normalised values, relative to the mean electricity consumption of February 2023.

**Fig. 3:**
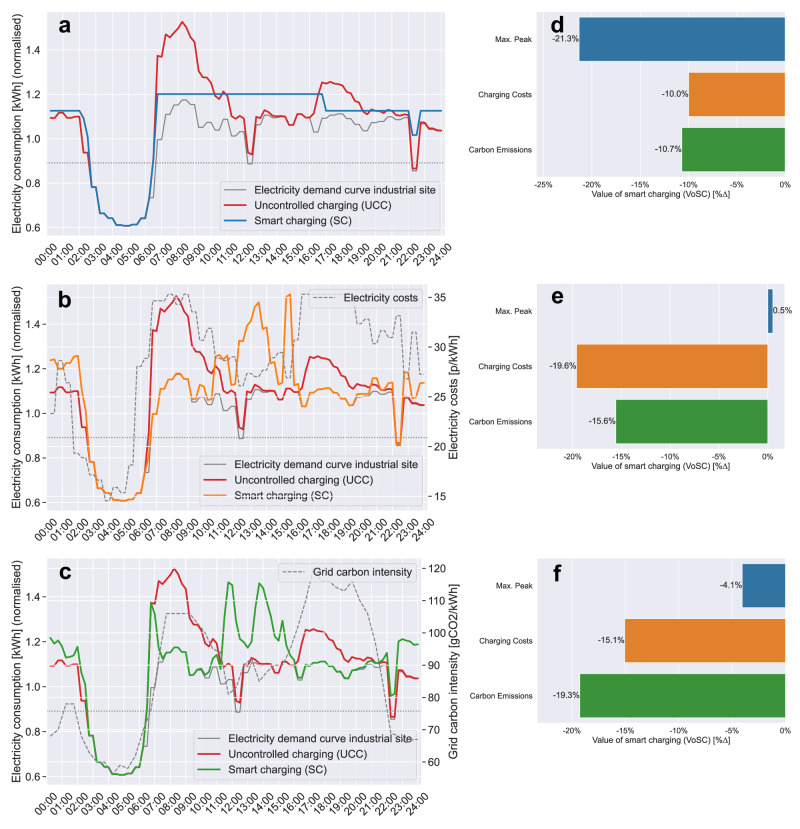
Charging profiles differentiated by model type for scenario 3 [S3: EV rate = 50%]. Time-series analysis of daily charging loads for (**a**) PM-VF, (**b**) CCM and (**c**) CEM. Quantitative assessment of relative change in output (VoSC, [%∆]) for each model type (**d**) PM-VF, (**e**) CCM and (**f**) CEM, measured against UCC, for the three key metrics max. peak demand [kWh], total charging costs [£] and total carbon emissions from charging [kgCO_2_].

For uncontrolled charging (UCC), the results, across all models, reveal that UCC leads to a substantial increase in electricity demand, coinciding with the pre-existing electricity demand peak during the early morning hours (06:00–09:00) as production activities ramp up. Due to more employee cars parked on-site during the AM shift, a higher charging demand during these hours leads to higher peak demand (Figs. 2–3). With an EV adoption rate of 15% [S1], amounting to 142 EVs, UCC leads to an 7.4% higher peak load compared to the previous maximum peak ($\max ({P}_{t})$) (Fig. 2). As more EVs are brought online, this effect exacerbates, leading to 29.9% higher peak loads for S3 (50% EV rate; 563 EVs) (Fig. 3); and 57.4% for S5 (100% EV rate; 1100 EVs) (Supplementary Fig. 4). This ‘dumb’ charging strategy serves as a reference to benchmark the outputs of the three objective functions against (Fig. 6).

When the PM-VF model is deployed, the optimiser computes a strategy that selectively schedules charging loads only during times of low overall electricity demand (*P*_*t*_), hence ‘filling local valleys’ with additional EV charging loads, as the objective function (eq. (2)) penalises larger deviations from the constant *C* (eq. (5)) (higher peaks) with a squared term.

Figure 2a shows that optimising for PM-VF for S1 (15% EV rate) does not incur any additional peak demand compared to $\max ({P}_{t})$, hence reducing peak demand by 7.4% compared to UCC (Fig. 2d). As the EV rate increases, the total charging demand grows to an extent where valley filling through load shifting does not suffice to meet the total demand, as shown in Fig. 3a (50% EV rate) and Supplementary Fig. 4a (100% EV rate), hence forcing the resulting electricity consumption profile to shift upwards. However, the resulting mostly flat total energy demand curve outperforms UCC in terms of reduced peak demand by 21.3% (Fig. 3d) and 28.5% (Supplementary Fig. 4d) for 50% and 100% EV adoption rates, respectively. Regarding charging costs and carbon emissions, PM-VF reduces total charging costs by 8.9%, 10.0% and 9.3% (Fig. 2d; Supplementary Fig. 4d) and total carbon emissions by 10.5%, 10.7% and 9.6% (Fig. 2d; Supplementary Fig. 4d) for EV rates of 15, 50, 100%, respectively. This is because total charging demand is evenly spread across times of high and low electricity prices and grid carbon intensity rates, in contrast to UCC where the majority of the charging load coincides with hours of high electricity prices and carbon-intensive grids.

**Fig. 4:**
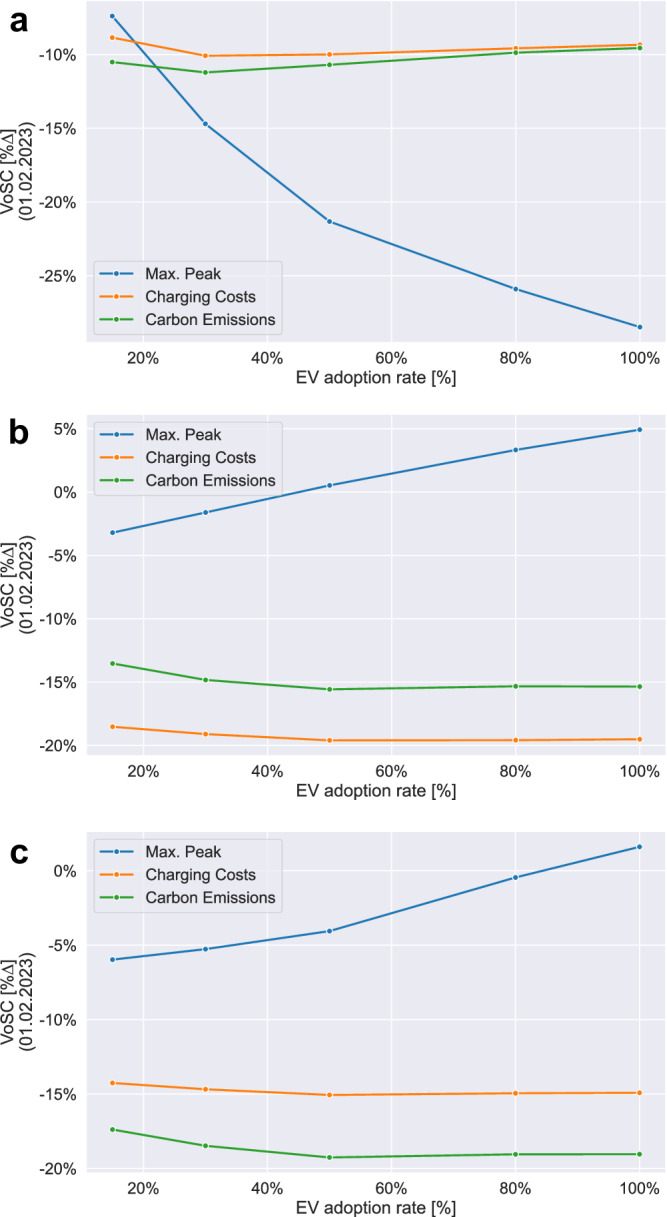
Visual summary of key metrics max. peak, charging costs and carbon emissions differentiated by model type for increasing EV rates [S1–5: 15–100%]. Quantitative assessment of output changes (VoSC, [%∆]), measured against UCC, for (**a**) PM-VF, (**b**) CCM and (**c**) CEM. Note: Lower %∆ numbers (y-axis) refer to higher saving potentials regarding each output metric.

Figure 4a summarises the outputs (*VoSC*[%∆]) obtained from PM-VF regarding the three main metrics maximum peak, total charging costs and resulting carbon emissions, including the two intermediary scenario cases of EV rates S2 = 30% and S4 = 80%. Results from the single-day analysis (01 February, 2023) show that model type PM-VF achieves substantially lower peaks with a reduction potential of up to 28.5%, while simultaneously reducing charging costs and carbon emissions by 9.3% and 9.6%, respectively, in a relatively stable manner across all five scenarios.

For model type charging cost minimisation (CCM), the optimiser shifts charging to times of low electricity prices (Fig. 2b), where the electricity price system used in our analysis is defined by the half-hourly Octopus Agile Time-of-Use (ToU) tariff ^31^, captured by the variable *λ*_*t*_. While this strategy leads to the lowest total charging costs, amounting to cost savings of about 19% and ~15% carbon emissions savings across all scenarios compared to UCC (Fig. 2b; Supplementary Fig. 4b), it becomes apparent that these gains are made by compromising on maximum peak demand reduction potential. Quantitatively, this trade-off between minimising for charging costs at the expense of higher peak demand intensifies as the EV adoption rate increases, from 3.2% of peak reduction potential in S1 (15% EV rate) (Fig. 2e) to an increase in maximum peak by 4.9% compared to UCC in S5 (100% EV rate) (Supplementary Fig. 4e). This relationship between minimising charging costs at the expense of increasing peaks for model type CCM is summarised in Fig. 4b.

For CEM—given the high correlation (*ρ* = 0.8) between charging costs *λ*_*t*_ and carbon emissions *γ*_*t*_ mainly due to carbon-intensive energy production methods (gas-fired power plants) during peak times—the results obtained for CEM turn out to be similar to CCM in terms of the overall trend. CEM yields carbon savings in the range of 17.4–19.3% across the various scenarios S1–S5 (EV rates: 15–100%) (Fig. 2f; Supplementary Fig. 4f). In contrast, optimising for overall lowest emissions increases total charging costs in direct comparison to CCM on average by about 4.5%-points. However, when measured against UCC, our results (*VoSC*[%∆]) suggest that total charging costs for CEM are still considerably lower by ~14.5% across all scenarios S1–S5 (EV rates: 15–100%) (Fig. 2f; Supplementary Fig. 4f).

When assessing the impact on maximum peak demand, the same trend of increasing peaks holds true for CEM as observed for model type CCM, however, not to the same extent. While CCM leads to substantially higher maximum peaks compared to UCC, in the case of CEM the maximum peak demand appears to be only slightly larger in magnitude (S5: 1.6%; EV rate: 100%) (Supplementary Fig. 4f). Fig. 4c visualises the trade-off between optimising for CEM and the resulting effect of increasing maximum peak demand as a function of various EV adoption rates (S1–5: EV rates 15–100%). All results (*VoSC*[%∆]) obtained from the scenario analysis with respect to varying EV adoption rates are reported in Supplementary Table 1 in numeric terms. In summary, we observe three threshold effects:

(1) For PM-VF, as the EV rate increases from 50% [S3] to 80% [S4], ‘valley filling’ as optimisation strategy to shift charging loads to times of lower overall electricity demand (*P*_*t*_) does not suffice anymore to meet the total EV charging demand. Hence, the resulting electricity consumption curve is shifted upwards (*P*_*t*_ + *y*_*t*_) which reduces the marginal savings potential (∆*VoSC*) [∆(%∆)] concerning the key metric maximum peaks, as shown by the flattening slope of the curve (Fig. 4a).

(2) For CCM and CEM, our results indicate a similar trend of decreasing marginal gains, i.e. savings potential in terms of lowering charging costs or total emissions, as the EV rate surpasses the threshold of 50% [S3] (Fig. 4b, c). This effect can be explained by a diminishing level of flexibility for the optimiser to shift charging loads to times of lowest electricity prices or low-carbon intensive grid conditions, as the EV rate rises. More precisely, the optimality space is further constrained by the increase in total charging demand (*y*_*t*_), as the binding condition pertaining to each EV to receive the requested charge (*E*_*T*+1_) before departure (cf. eq. 12) becomes more restrictive.

(3) Lastly, for both CCM and CEM, rising EV adoption is associated with increasing maximum peaks, even to the extent of exceeding UCC-levels [S5]. The effect size of this observed relationship is greatest at the threshold between EV rate = 50% [S3] and 80% [S4] (Fig. 4b, c). This exhibited trend can be traced back to the nature of a single objective model formulation. In practical terms, this means that the optimiser schedules charging processes to take place during the most optimal times of low electricity prices or low carbon-intensive grid conditions while adhering to all other constraints, for instance limitations on physical charge point infrastructure related to maximum charging power rate [kW] (cf. eq. 14), without considering any other possibly desirable secondary objective. Hence, this results in a trade-off space, unless a more advanced bi-objective modelling approach is implemented.

### Temporal sensitivity analysis

Building upon prior results presented for a single-day analysis (01 February, 2023) adopting the structure of three objective functions measuring three key output metrics for five EV adoption scenarios, we now apply the same methodological structure to a multi-day analysis spanning 28 consecutive days in February 2023 as empirical focus. Augmenting the unit of analysis from a single-day to a month-long time horizon, encompassing 28 individual model runs, allows us to measure the effects of varying exogenous time-dependent input parameters on the overall sensitivity of the model results. These parameters include (i) the industrial site’s electricity consumption curve (*P*_*t*_), (ii) the ToU electricity tariff (*λ*_*t*_), and (iii) the grid carbon intensity profile (*γ*_*t*_) (cf. Fig. 6). Ultimately, these insights help us assess the generalisability of the prior single-day analysis based on real-world empirical data, allowing us to derive conclusions concerning the overall robustness of the model results.

**Fig. 5:**
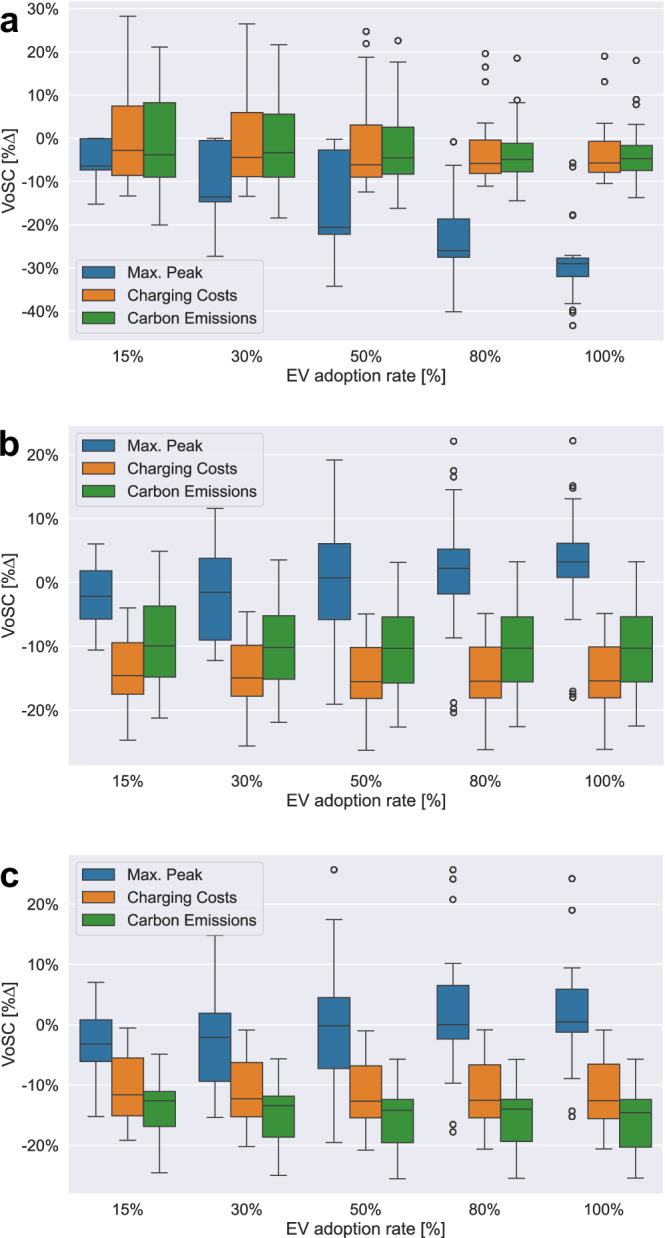
Overview of model results, grouped by model type, for increasing EV rates [S1–5: 15–100%], computed over a 4-week long time frame [Feb 2023]. Statistical analysis of 28 single-day model results, capturing output changes (VoSC, [%∆]), measured against UCC, for each model type (**a**) PM-VF, (**b**) CCM and (**c**) CEM by plotting the variability of the key output metrics (i) max. peak (blue), (ii) charging costs (orange) and (iii) carbon emissions (green) using boxplots as visualisation tool. Note: Lower %∆ numbers (y-axis) refer to higher saving potentials.

Figure 5 provides an overview of the results obtained from the temporal sensitivity analysis for (a) PM-VF, (b) CCM and (c) CEM, in each case visualising the variability of daily output measures pertaining to the three key metrics maximum peak (blue), charging costs (orange), and carbon emissions (green) for all five EV adoption rate scenarios [S1–5: 15–100%].

Drawing from the results, we derive two main insights:

(1) On average, the respective key output metric of each model type, i.e. maximum peak for PM-VF, charging costs for CCM and carbon emissions for CEM, exhibits the highest absolute output changes (∣%∆∣), i.e. largest absolute savings potential, when benchmarked against UCC, across all EV adoption rate scenarios (Fig. 5a–c). This indicates that each model type achieves its respective objective by computing efficient charging strategies aligned to the respective goal of peak-, charging costs- or emissions minimisation.

(2) Contrary to model types CCM and CEM which yield output results that are fairly constant (Fig. 5b, c), i.e. exhibiting low levels of variability across EV adoption rate scenarios, we observe that for PM-VF variability decreases substantially as the EV rate increases, hence making the outcome more predictable (Fig. 5a). This trend applies to all three key output metrics, most prominent in terms of maximum peak. Visually, this can be observed from the reduction in length of the interquartile range (IQR) of the respective boxplots.

In summary, while our analysis shows that each model type achieves optimal results for its specific objective by computing efficient charging strategies, it also reveals that trade-offs between individual goals are inevitable.

## Discussion

Expanding the existing charge point infrastructure to fuel the transition to an electrified transportation system remains an ongoing challenge. With the predicted accelerated uptake of EVs among wider adopter groups^32^, moving from early adopters to the early majority^33^, there is mounting pressure on firms to invest in the expansion of EV charge points in their employee car parks. According to a recent industry report by ChargeUK, the number of workplace chargers is expected to increase five-fold by 2030 in order to keep pace with the predicted EV adoption rates^34^.

Planning the expansion of charging points, along with the associated technical and economic challenges, has predominantly been the responsibility of local and regional network operators. Consequently, the capacity for individual firms to conduct predictive impact assessments of the additional loads from EV charging has thus far been significantly limited, primarily due to a lack of internal energy management capabilities. Similarly, we identified a gap in academic literature that addresses the role and responsibilities of individual firms to engage in planning activities related to the build-out of EV workplace charging infrastructure (cf. Introduction).

Our research addresses this gap by taking on the perspective of workplace operators as key agents in planning the transition to a low-carbon energy system with close interactions with the mobility sector. We show that large-scale, uncontrolled deployment of EV workplace charging can cause severe system inefficiencies, leading to significantly higher peaks, increased charging costs and greater carbon emissions. Conversely, the use of advanced control mechanisms and well-orchestrated SC strategies can result in substantial savings, offering several co-benefits in economic and environmental sustainability terms.

The results obtained from the temporal sensitivity analysis indicate that the deployed models yield robust outcomes to time-variant parameters, repeatedly outperforming UCC in the respective key metrics. However, we also show that in the case of CCM and CEM, a single-objective model formulation can jeopardise the relative performance of the respective other metrics, such as maximum peak, which are not jointly optimised for, once the EV adoption rate exceeds a certain threshold. This highlights that trade-offs between various objective functions and the associated outcome metrics are inevitable. We derive these insights based on iterative testing of the models in changing environments, as evidenced in the real world.

Abstracting from the case study presented, we argue that the method of quantifying estimates of savings potential incurred from deploying SC solutions, measured in economic and environmental sustainability terms, is transferable to other contexts of EV charging in which passenger cars are parked for an extended period. This applies, for instance, to non-industrial workplace settings (office buildings only), workplaces in other sectors (hospitals, universities), or in settings of mid- to long-duration destination parking (gym, cinema, train stations, airports etc.). Generalising from our study, we infer that the *VoSC*[%∆] hinges on (i) the seasonality and location-based variability of grid carbon intensity levels, (ii) market-based price signals induced from different ToU electricity tariffs, (iii) key modelling assumptions related to EVs’ SoC levels, (iv) the ratio of total number of EVs, their respective SoC levels, in direct relation to the site’s total electricity consumption levels (*P*_*t*_) and closely related, (iv) the general shape of the site’s electricity demand curve.

In summary, our study highlights the complexity of planning the low-carbon transition to electrified passenger transportation in the context of EV workplace charging. We show that this process necessitates a multi-faceted approach that combines future EV electricity demand scenario planning with firm-specific cost- and carbon-budgeting, as well as grid-planning activities.

Given certain restrictions concerning the mathematical formulation of the core optimisation problem and limited access to real-world data, the model exhibits several limitations. First, the reduction in charging power from constant current to constant voltage (CC-CV) once a certain battery charge level has been reached (typically SoC ≥ 80%)—a technical requirement from an electrical engineering point of perspective—is not reflected in the optimisation tool. Hence, the total charging duration to reach the final 20% SoC level takes longer in reality. However, given that the primary focus of the optimisation tool is to empower business executives to assess the broader implications of EV workplace charging for their firm-specific metrics, we argue that the current version of the tool delivers these high-level planning insights to a satisfactory degree. Second, given that there are no industrial ToU electricity tariffs available in the UK at the point of writing, we opted for the Octopus Agile tariff, which is available to residential customers. Third, we acknowledge certain limitations with respect to model parametrisation, including assumptions regarding the distribution of battery SoC levels upon arrival and minimum charge requirements upon departure due to a lack of real-world data from the collaborating partner. Fourth, charging profiles in this study are simulated, not based on real-world observations, given that the manufacturing firm, our case study partner, was still at the planning and contracting stage and had not yet installed charging stations. We chose not to draw on static, non-flexible EV charging loads from real-world EV trials due to the particular study design which necessitated SC loads to be modelled endogenously, allowing for sufficient degrees of freedom to schedule EV-specific charging cycles by factoring in (i) EV-specific arrival and departure times, (ii) firm-specific exogenous electricity consumption patterns and (iii) external, market-driven signals, including time-varying electricity prices and grid-carbon intensity levels. This allowed us to assess the firm-specific demand-side flexibility potential arising from SC, respective to three different charging strategies. Fifth, we acknowledge that due to limited provision of electricity demand data from our case study partner, covering February–June 2023 only, we were unable to perform a whole year temporal sensitivity analysis. However, it is important to note that electricity infrastructure is designed to operate within a specified margin, determined by the size of demand that piece of infrastructure is serving at peak conditions^35^, which in the UK and Northern Europe usually occurs on winter weekdays. This period sees increased private car use due to reduced active travel in colder temperatures^36^ and peak electricity demand from heating, lighting and industrial activities^37^. Hence, we chose February 2023 to compute our results across three metrics and for three objective functions to be able to provide relevant insights for a reference month in which critical peak demand is observed.

Beyond validating the model with real-world mobility data, we identify opportunities for further research in the following areas: First, the model could be expanded to include a charge point allocation algorithm based on priority-based ruling, for instance, for EVs with lower initial SoC levels. Second, enhancing the web app to include an economic cost-benefit decision support mechanism for determining the optimal number of EV charge points to install represents another avenue for further research. Third, expanding the system boundaries would allow for the evaluation of how varying degrees of employee access to charge points outside the workplace, as well as users’ preferences for charging locations in relation to specific tariff structures, impact the firm’s overall electricity demand. Lastly, evaluating the implications of incorporating bidirectional (Vehicle-to-Grid, V2G) charging capabilities into the existing model framework could provide additional valuable insights.

## Methods

This section is structured as follows: First, we introduce the overall modelling framework. Second, we describe the methodological approaches pertaining to parametric assumptions and time-series data used for the case study. Third, we outline the complete mathematical model formulation, including a full list of constraints and nomenclatures of variables, sets and parameters. Fourth, we elaborate on the choice of threshold values relevant for the prospective ‘what-if’ scenario analysis.

### Modelling framework

**Fig. 6:**
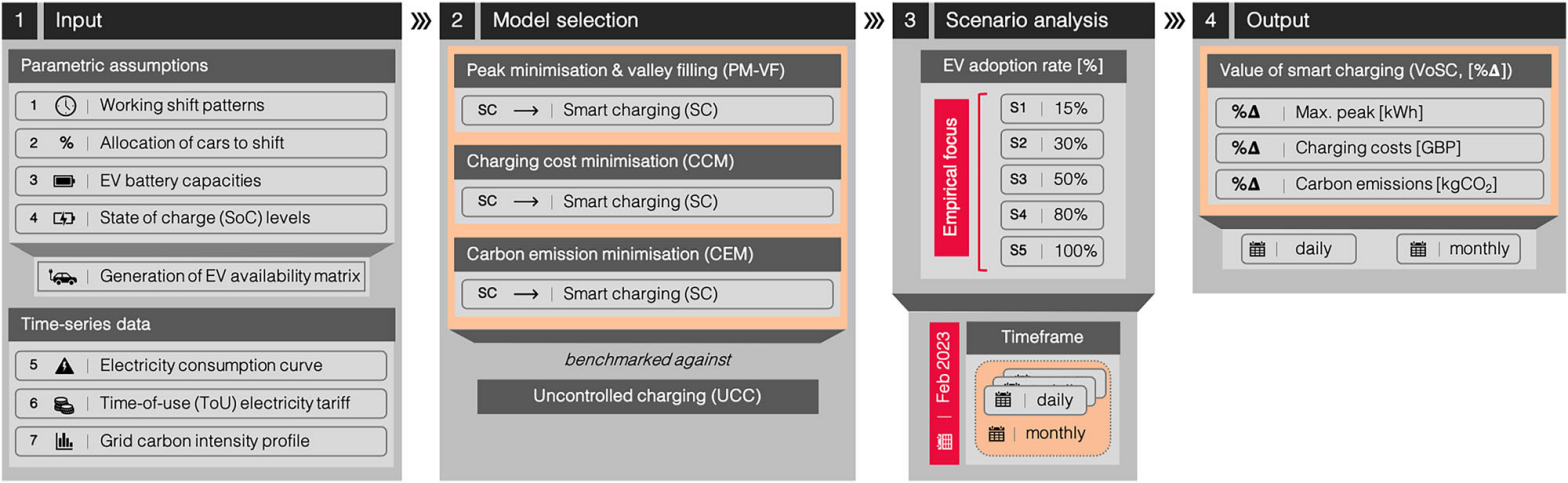
Schematic overview of our modelling framework. **Step 1** Specification of input parameters, including time-series data. **Step 2** Selection of model, assessing (i) peak minimisation & valley filling (PM-VF), (ii) charging cost minimisation (CCM), or (iii) carbon emission minimisation (CEM) against uncontrolled charging (UCC). **Step 3** Scenario analysis with varying EV adoption rates [%] on a daily & monthly scale. **Step 4** Computation of model results for each objective function, incorporating parametric and temporal scenario analysis, benchmarked against UCC in relative terms (VoSC, [%∆]).

Our study quantifies the impact of EV workplace charging on an industrial site’s electricity demand profile based on three different optimisation goals. To this end, we developed a modelling framework, outlined in Fig. 6, that consists of four steps:

First, we input parametric assumptions specifying (1) the number of working shift patterns with their respective start and end times, (2) the total number of vehicles coming to the workplace each day with a relative distribution [%] assigned to each shift, (3) the considered EV battery capacity sizes and (4) assumptions regarding initial and final SoC levels, the latter being specified by EV users as minimum charge expectation. Subsequently, an EV parking availability matrix is generated which serves as core modelling input, alongside time-series data specifying the (5) electricity demand profile of the industrial site, (6) a ToU electricity tariff and (7) the grid carbon intensity profile, all in 15-min time intervals.

Second, we select the respective model of interest, each differing in terms of model objective: (a) grid performance objective: PM-VF, (b) economic objective: CCM, and (c) environmental objective: CEM. Each model is assessed against an UCC strategy, in which the charging process starts upon plug-in at arrival, without the flexibility of load shifting.

Third, with a fixed EV availability matrix, we then compute a scenario analysis, measuring changes to the output (in %∆) by varying the EV adoption rates according to five scenarios [S1–5], ranging from 15% to 100% [S1–5: 15, 30, 50, 80, 100%].

Fourth, and lastly, we augment the single-day analysis to a monthly analysis by iterating over each day in a respective month (for this case study: February 2023), hence capturing the changes incurred from varying time-series inputs (electricity consumption profile of industrial site, ToU electricity tariff, grid carbon intensity profile). All outputs obtained from the sensitivity analyses are reported in percent-changes [%∆], measured against the UCC strategy for each EV adoption rate scenario [S1–5].

### Input parameters

For the case study presented, we analysed a manufacturing environment that operates on a two-gear shift pattern for employees working in assembly, in addition to a single shift for office staff. We estimated the total number of vehicles in the parking lot at 1100 per day, with a relative allocation of 63%, 27% and 10% of all cars to each shift (AM, PM, office), respectively.

Catering for different EV model types, we assumed battery capacity sizes to be uniformly distributed according to three levels (48, 71, 100 kWh) which we derived based on expert elicitation and current EV manufacturing market trends. To model heterogeneity in charging demand, we modelled initial SoC levels (${E}_{m}^{ini}$) of each EV’s battery upon arrival at the workplace to be randomly distributed between 10% (lower bound, LB) and 80% (upper bound, UB), and similarly between 80% (LB) and 100% (UB) as minimum SoC requirement (${E}_{m}^{fin}$) upon departure, to be specified by the respective EV user. While these parametric assumptions have been chosen to reflect the case study of analysis, the open-source web application (cf. Supplementary Fig. 1) allows for flexible input data entry to assist practitioners in developing bespoke scenarios, to be aligned with firm-specific real-world settings.

Based on the specified input data, an array of agents representing individual EVs with their associated parameters (arrival and departure times, EV battery capacity sizes, SoC levels) is generated. Depending on the scenario selected, the binary EV availability matrix, indicating whether the respective EV *m* ∈ *M* is parked and available for charging at time step *t* ∈ *T* (step size: *τ* = 15 min) (*f*_*mt*_ = 1), or not (*f*_*mt*_ = 0) (cf. eq. (8)), varies in number of entries, ranging from 142 [S1: 15% EV rate] to 1100 [S5: 100% EV rate]. While S1 has been calibrated to represent the real-world setting of the global car manufacturer in terms of actual EV adoption rate (15%) in 2023, for S5 we assume a future scenario in which all 1100 employee cars are fully electrified (EVs).

Subsequently, the exogenous time-series data pertaining to each firm’s bespoke electricity consumption profile *P*_*t*_, in addition to the location-dependent market-based ToU electricity tariff *λ*_*t*_ (Octopus Agile Tariff^31^) and UK-wide grid carbon intensity profile *γ*_*t*_ (NationalGridESO^38^) is inputted, either via manual .csv-file upload (*P*_*t*_; cf. Supplementary Fig. 1) or pulled via API integration (*λ*_*t*_; *γ*_*t*_) based on the date(s) specified. Supplementary Fig. 5 displays these exogenous time-series data for *P*_*t*_, *λ*_*t*_ and *γ*_*t*_.

In summary, our study uses a mix of real-world data, combining (i) firm-specific data from a global car manufacturer (electricity consumption profile [kWh], work shift pattern, number of employee cars parked on-site during each shift), (ii) general market data (Octopus Agile ToU tariff, grid carbon intensity profile, selected EV battery sizes), in addition to (iii) simulated assumptions (EVs’ SoC levels upon arrival and departure, EV adoption rate scenarios [S1–5; %]).

### Formulation of mathematical optimisation problem

Drawing from operations research methods on mixed-integer linear programming and quadratic optimisation, the core module of this study builds upon prior work by Ioakimidis et al.^29^ and Zheng et al.^30^. We define three objective functions, each representing a different model:

- Peak minimisation & valley filling (PM-VF)
  2 $$min.\quad {z}_{PM-VF}=\sum _{t\in T}\,{({P}_{t}+{y}_{t}-C)}^{2},$$
  where *z*_*PM*−*VF*_ captures the objective function value, *P*_*t*_ refers to the industrial site’s electricity demand profile, *y*_*t*_ is the total load from EV charging and *C* is a constant (eq. (5)).
- Charging cost minimisation (CCM)
  3 $$min.\quad {z}_{CCM}=\sum _{t\in T}\,{y}_{t}* {\lambda }_{t},$$
  where *z*_*CCM*_ captures the total charging costs and *λ*_*t*_ refers to the real-time electricity prices, as set by the ToU electricity tariff.
- Carbon emission minimisation (CEM)
  4 $$min.\quad {z}_{CEM}=\sum _{t\in \,T}\,{y}_{t}* {\gamma }_{t}.$$
  where *z*_*CEM*_ captures the total carbon emissions and *γ*_*t*_ represents the marginal carbon emissions rate, also referred to as the grid carbon intensity, per kWh.
  Also, note the following:
  5 $$C=\frac{\max ({P}_{t})+\min ({P}_{t})}{2},$$
  6 $${y}_{t}=\sum _{m\in M}{x}_{mt}* {f}_{mt}\quad \forall t\in T,$$
  7 $$0\le \,{x}_{mt}\le \,{p}_{max}\quad \forall m\in M,t\in T,$$
  where *x*_*mt*_ is the core decision variable (DV), defined as non-negative real number ${x}_{mt}\in {{\mathbb{R}}}_{\ge 0}$ and capped by the maximum charging power rate *p*_*max*_ as upper bound (eq. (7)), that captures the charging profile for each EV *m* ∈ *M*, for all *t* ∈ *T*. Furthermore, *f*_*mt*_ represents the exogenous (binary) EV availability matrix (eq. (8)), defined as:
  8 $${f}_{mt}=\left\{\begin{array}{l}1, \,\,{\rm{if}}\, {\rm{EV}}\,m\in M\,{\rm{is}\, {\rm{parked}}\, {\rm{at}}\, {\rm{the}}\, {\rm{workplace}}\, {\rm{at}}\, {\rm{time}}}\,t\in T\\ 0, \,\,{\rm{otherwise}}\,.\end{array}\right.$$

In each model, the optimiser computes the optimal charging profile for each EV *m* ∈ *M*, where *M* refers to the total set of EVs, assuming perfect foresight of each car’s parking duration and stochastic arrival and departure times.

Each objective function (eq. (2)–(4)) optimises towards a certain goal. For PM-VF, the model draws on techniques from quadratic optimisation to compute an efficient charging strategy that reduces overall peaks, hence, aiming to minimise the least-square difference between the sum of the fixed electricity demand profile *P*_*t*_ and the variable EV charging load *y*_*t*_ (DV) at each time step *t* and a constant *C*, defined as the average value of *max*(*P*_*t*_) and *min*(*P*_*t*_) on a daily basis (eq. (5)). For CCM and CEM, the model takes on the form of a mixed-integer linear programme (MILP) with the objective of minimising total charging costs (CCM) or total carbon emissions (CEM) by computing the sum product of total charging demand *y*_*t*_ (DV) and *λ*_*t*_, the ToU electricity price tariff, or *γ*_*t*_, the grid carbon intensity profile. The results of each optimisation task are stored in the respective objective function variable *z*_*PM*−*VF*_, *z*_*CCM*_ and *z*_*CEM*_ which constitutes the core output data for the three key metrics max. peak demand [kWh], total charging costs [£] and total carbon emissions [kgCO_2_]. These model results are subsequently benchmarked against UCC, in which case charging of EVs *m* ∈ *M* is assumed to initiate directly after plug-in upon arrival at the workplace, by computing the VoSC (eq. (1)).

For all models (eq. (2)–(4)), several constraints ensure that (i) charging only takes place while EVs are being parked at the workplace, (ii) the final battery SoC levels, as requested by each EV user, are achieved and (iii) total battery capacity is not exceeded. A complete overview of the full model formulation is provided in Table 1.

**Table 1:** Complete model formulation including objective functions and constraints

Model type		PM-VF	CCM	CEM	eq.
Obj. function	*min*.	$z={\sum }_{t\in T}\,{({P}_{t}+{y}_{t}-C)}^{2}$	*z* = ∑_*t*∈*T*_ *y*_*t*_$\,*\,$λ_*t*_	*z* = ∑_*t*∈*T*_ *y*_*t*_$\,*\,$γ_*t*_	(9)
Constraints	*s*. *t*.	*y*_*t*_ = ∑_*m*∈*M* _*x*_*mt*_ $*$ *f*_*mt*_		∀ *t* ∈ *T*	(10)
		$0\le \,{E}_{m}^{ini}+{\sum }_{k\in T | k\le t}\tau * {x}_{mt}* {f}_{mt}\le \,{E}_{m}^{cap}$		∀ *t* ∈ *T*; *m* ∈ *M*	(11)
		${E}_{m}^{fin}={E}_{m}^{ini}+{\sum }_{k\in T | k\le t}\tau * {x}_{mt}* {f}_{mt}\ge {E}_{T+1}$		∀ *t* ∈ *T*; *m* ∈ *M*	(12)
		0 = *x*_*mt*_$\,*\,$(1 − *f*_*mt*_)		∀ *t* ∈ *T*; *m* ∈ *M*	(13)
		0 ≤ *x*_*mt*_ ≤ *p*_*max*_		∀ *t* ∈ *T*; *m* ∈ *M*	(14)
where,		$C=\frac{\max ({P}_{t})\,+\,\min ({P}_{t})}{2}$			(15)
and		$${f}_{mt}=\left\{\begin{array}{l}1,\quad \,{\text{if}}\,{\text{EV}}\,\,{\text{m}}\in {\text{M}}\,{\text{is}}\, {\text{parked}}\,{\text{at}}\,{\text{the}}\,{\text{workplace}}\,{\text{at}}\,{\text{time}}\,\,t\in T\\ 0,\quad \,{\text{otherwise}}\,.\end{array}\right.$$			(16)

Overview of quadratic optimisation (PM-VF) and mixed-integer linear programme (CCM/CEM).

Source: Adapted from refs. ^29^^,^^30^.

Each constraint serves a distinct purpose: Eq. (10) captures the total charging load at each time step *t* ∈ *T* in a dedicated auxiliary variable (AV) *y*_*t*_ by summing over all individual EVs *m* ∈ *M*. Eq. (11) defines the boundary conditions, enforcing that charging stays within the physical limits of the battery capacity size of each EV *m*. Similarly, eq. (12) ensures that the minimum charge requirement, specified by the EV user and captured in the exogenous parameter *E*_*T*+1_, is delivered prior to departure. The resulting battery charge level is subsequently stored in the AV ${E}_{m}^{fin}$. Eq. (13) is a logic operator, linking *x*_*mt*_ with the binary EV availability matrix *f*_*mt*_ (eq. 16) in a way that forces *x*_*mt*_ = 0 whenever EV *m* at time step *t* is not parked at the workplace (*f*_*mt*_ = 0). Finally, eq. (14) defines the range of DV *x*_*mt*_, limiting charging per time interval *t* to stay within the limits of the maximum charging power rate of the respective hardware charge point infrastructure (11 kW for our case study).

In summary, Table 2 provides an overview of the nomenclature by listing all sets, parameters and variables used throughout the mathematical model.

**Table 2:** Nomenclature

*Sets*	
*M* = {*m*}	Set of EVs, where *m* represents a single EV
*T* = {*t*}	Set of time slots, where the duration of a time slot *t* is fixed and given
*Parameters*	
*P*_*t*_	Electricity demand curve of industrial site at time step *t* [kWh]
*λ*_*t*_	Time-of-Use (ToU) electricity price tariff at time step *t* [p/kWh]
*γ*_*t*_	Grid carbon intensity at time step *t* [gCO_2_/kWh]
${E}_{m}^{cap}$	Total battery capacity of EV *m* [kWh]
${E}_{m}^{ini}$	Initial battery charge level of EV *m* upon arrival at workplace [kWh]
*E*_*T*+1_	Minimum battery charge requirement after work shift (specified by EV user) [kWh]
*p*_*max*_	Maximum charging power capacity of charge point [kW]
*f*_*mt*_	Parking availability matrix (binary) of EV *m* indicating arrival and departure times
*C*	Average value of daily max. and min. *P*_*t*_ [kWh]
*τ*	Length of each time interval *t* [15 min]
*Auxiliary variables (AVs)*	
*y*_*t*_	Total electricity demand from EV charging at time step *t* [kWh]
${E}_{m}^{fin}$	Final battery charge level of EV *m* upon departure from workplace [kWh]
*Decision variable (DV)*	
*x*_*mt*_	Charging electricity demand for EV *m* at time step *t* [kWh]

### Scenario development

We define five different scenarios (S1–S5) that capture the degree of EV adoption [%] relative to the total stock of employees’ cars. To this end, we chose specific threshold values [S1–S5: 15, 30, 50, 80, 100%] that mark crucial points along the S-curved technology adoption pathway^33^. It is predicted that by ~2055, 100% of all vehicles in the UK will be electric, with a rapid transition from 20% to 80% occurring in just 8 years between 2033 and 2041^32^. More in-depth insights concerning EV adoption pathways on a local, highly spatially granular level can be derived from a dedicated open-source ‘S-Curve Adoption Tool for EVs’ (SCATE), developed by the Energy and Power Group at the University of Oxford.

## Supplementary information

Supplementary information

## Data Availability

The datasets analysed during the current study are not publicly available due to commercial sensitivity but are available from the corresponding author on reasonable request.

## Code availability

The programming source code was written in Python using the Pyomo^39^ library for mathematical optimisation. It is accessible on GitHub via https://github.com/segermarcel/EVWorkplaceChargingPublic.
