# Supplementary material for: Firm level optimisation strategies for sustainable and cost effective electric vehicle workplace charging
Source: NPJ Sustain Mobil Transp. 2025 Mar 17;2(1):11. doi: 10.1038/s44333-025-00032-w (PMC11913721; doi:10.1038/s44333-025-00032-w)
Supplement: Supplementary file 1 — Supplementary information [file 44333_2025_32_MOESM1_ESM.pdf]

Supplementary Information

Development of open-source web application

Beyond the case study discussed, we present ongoing work on developing an interactive, open-source web application. The goal of this tool is to empower business executives, particularly those with limited internal capabilities, to take control over energy management, enabling them to adequately plan and operate EV workplace charging infrastructure according to bespoke scenarios. The tool provides highly detailed temporal and spatial insights into the resulting electricity consumption profile according to three charging strategies: Peak minimisation & valley filling (PM-VF), charging cost- (CCM), and carbon emission minimisation (CEM). We tested the web app based on real-world data from a large car manufacturing firm in South East England, UK.

The tool is structured as follows: The sidebar on the left serves as input for the exogenous parameters while the remaining page outputs the core results for the single-day analysis, consisting of line plots and bar charts, each of which summarise the outcomes of the three charging strategies (PM-VF, CCM, CEM) in accordance with the pre-selected EV adoption rate scenario (%). Supplementary Figure 1 shows the current version of the EV workplace charging web application. In terms of system architecture, the tool is built in a modular manner, combining Pyomo's [1] libraries for mathematical optimisation with Streamlit's [2] functionalities for web app development. It is hosted on the Streamlit community cloud and publicly accessible via https://ev-workplace-charging.streamlit.app/ for use with own data. The original source code can be reviewed in GitHub.

The tool takes the following parameters as input: (i) start and end times of work shifts, including number of cars parked and available for charging; (ii) common battery capacity sizes of employees' EVs, including the relative portion of each battery type; (iii) electricity demand curve of industrial site (.csv-file upload); (iv) date of interest; (v) solver type; (vi) charger output power rate and (vii) EV adoption rate (%). Then, an EV availability matrix is generated with varying state-of-charge (SoC) levels, to be uniformly distributed between a lower- (LB) and an upper bound (UB) (% of total EV battery capacity) (refer to Methods section for more granular insights on main assumptions used in our case study). Depending on the date selected, the respective time-of-use (ToU) electricity price data (Octopus Agile Tariff [3]) and the UK-based grid-carbon intensity [4] are automatically pulled via their respective Application Programming Interface (API). By default, the Gurobi solver [5] is used to compute cost-effective and sustainable charging strategies.

Applying this tool in a real-world setting based on bespoke firm-specific data allows practitioners to plan the low-carbon transition to electrified mobility in a much more spatially granular manner. Building upon the case study presented, we encourage more businesses to actively consider the implementation of digital software solutions, e.g., by using our open-source web app, for managing EV charging loads as an equal counterpart to the roll-out of charging hardware infrastructure. By leveraging state-of-the-art optimisation techniques, that are made accessible via a practical, easy-to-use tool, firms can reduce the dependency on large build-outs of electricity grid infrastructure, while increasing the share of renewable energy sources fed into the grid.

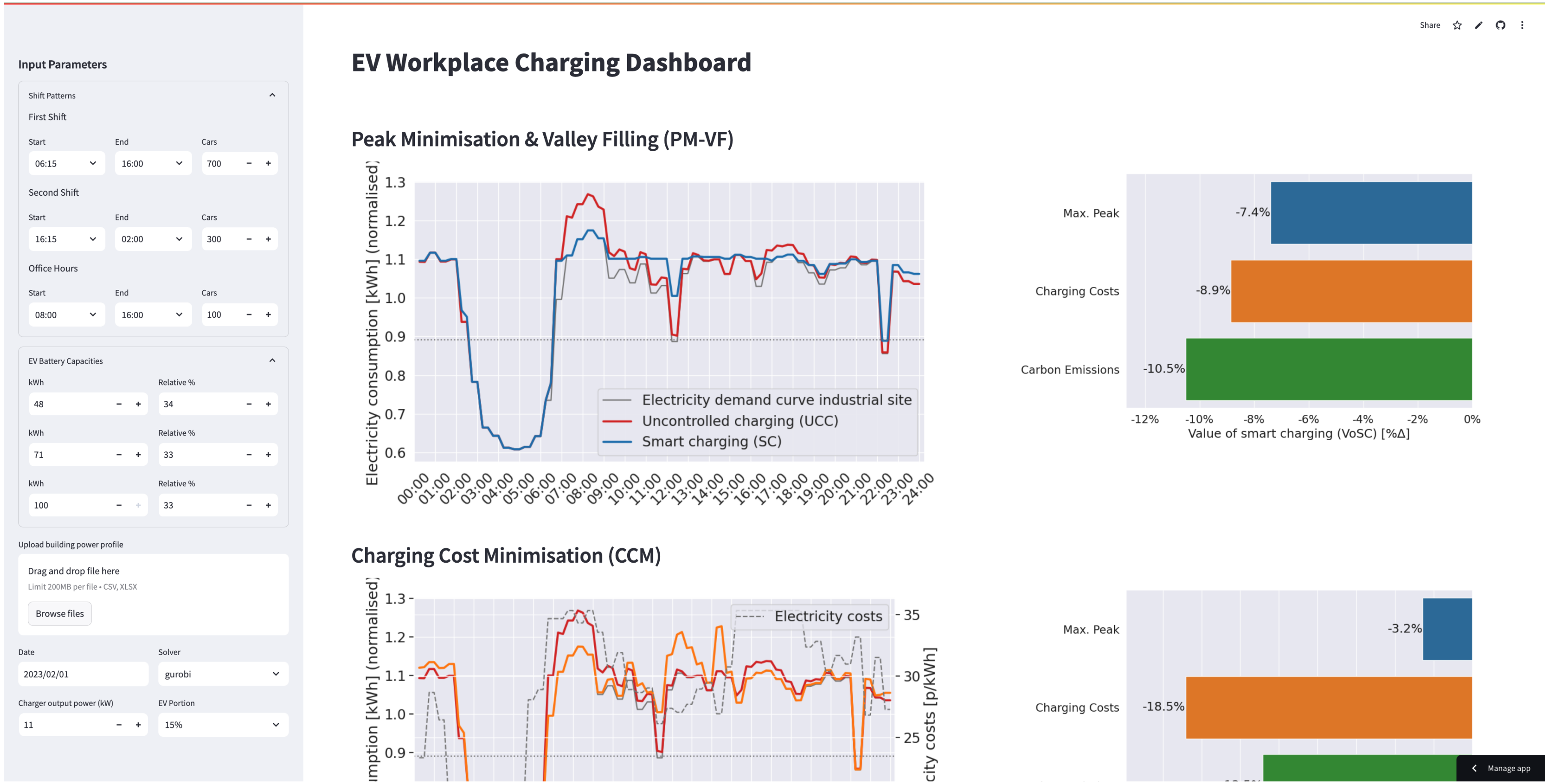

Supplementary Figure 1 | **User interface of EV workplace charging web application.** The interactive tool can be accessed via https://ev-workplace-charging.streamlit.app/, deployed and made publicly available (open-source) on Streamlit's community cloud service.

Supplementary Figures and Tables

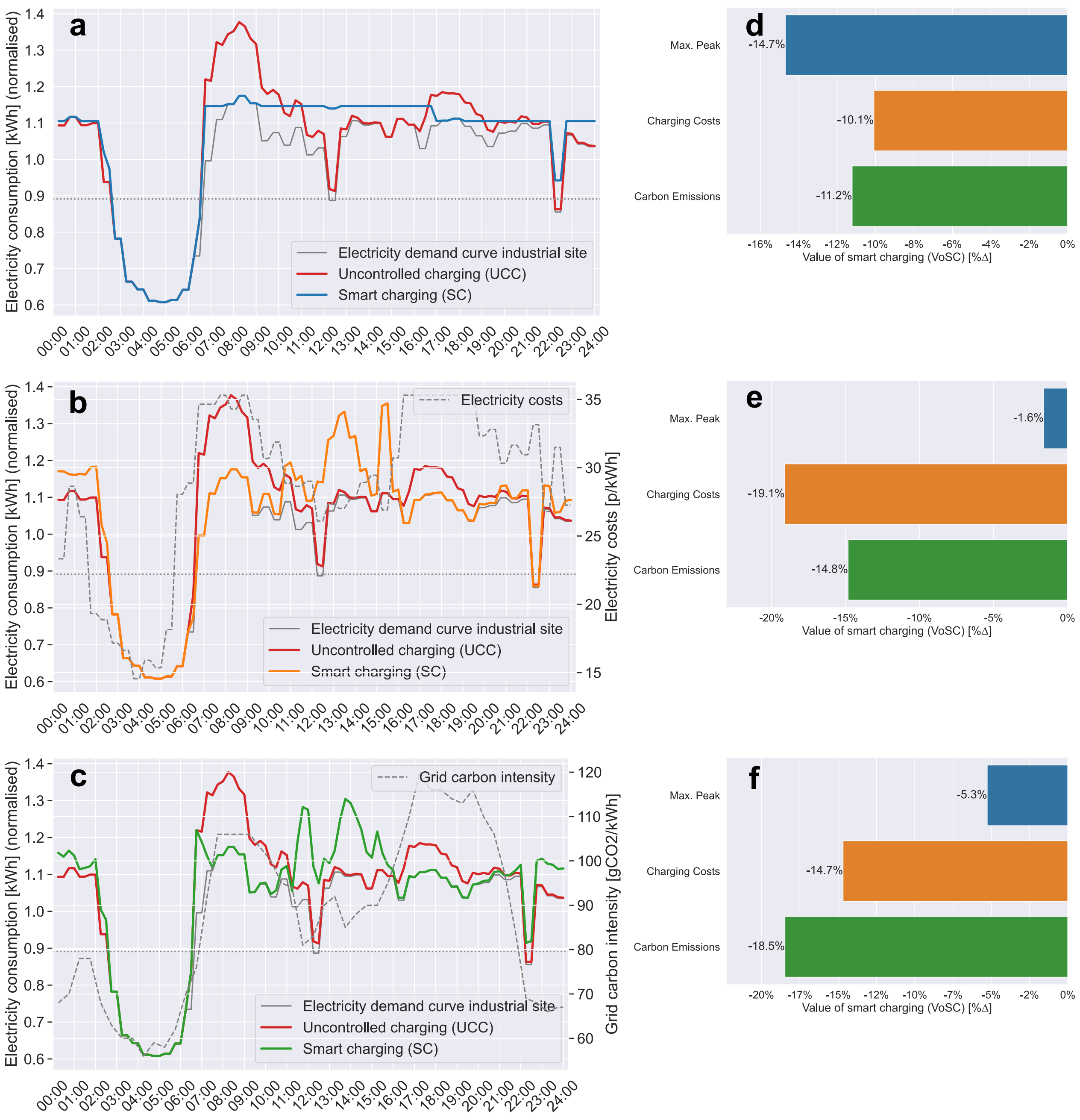

Supplementary Figure 2 | Charging profiles differentiated by model type for scenario 2 [S2: EV rate=30%]. Time-series analysis of daily charging loads for **(a)** PM-VF, **(b)** CCM, and **(c)** CEM. Quantitative assessment of relative change in output (VoSC, [%Δ]) for each model type **(d)** PM-VF, **(e)** CCM, and **(f)** CEM, measured against UCC, for the three key metrics max. peak demand [kWh], total charging costs [£], and total carbon emissions from charging [$kgCO_2$].

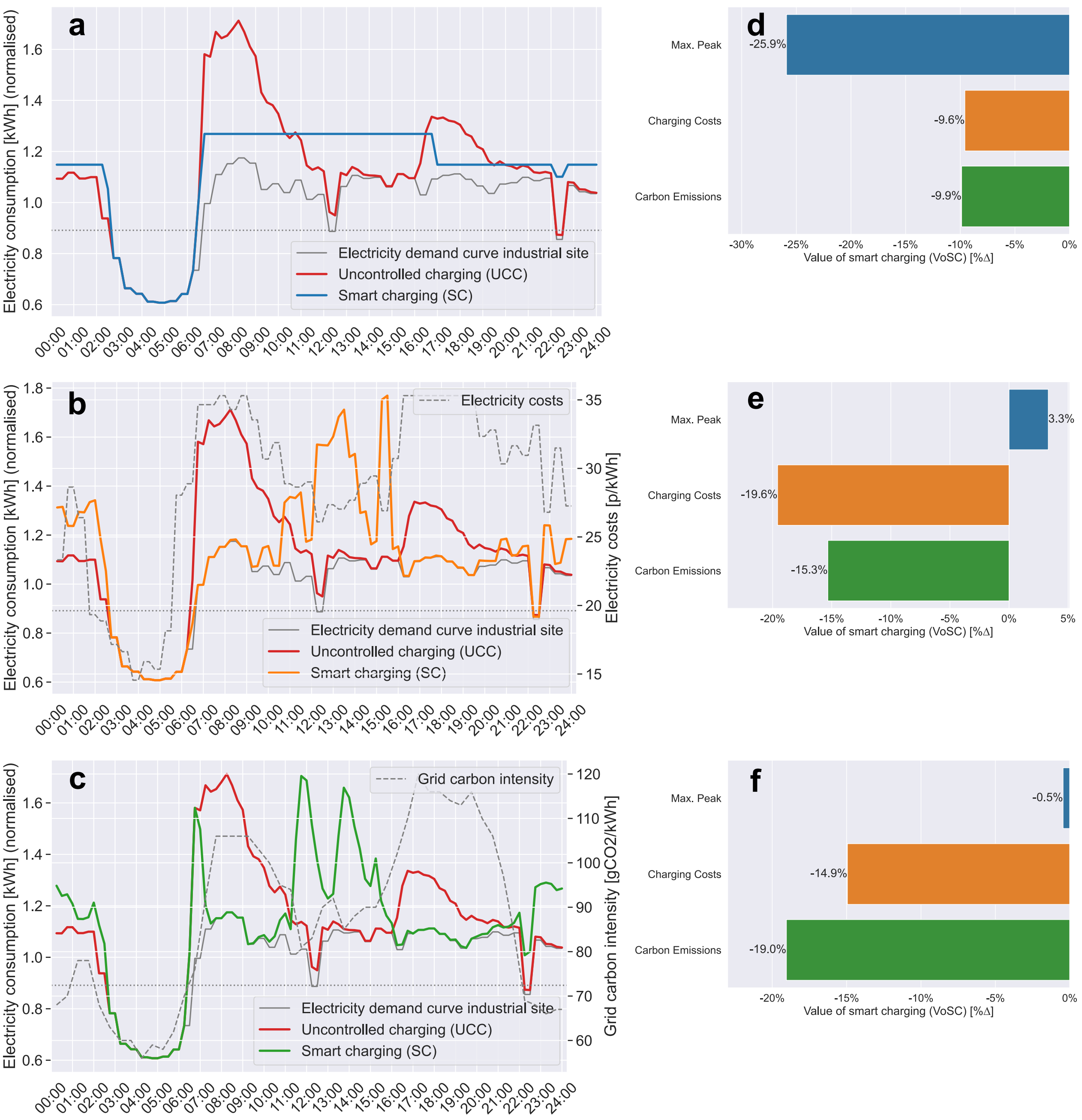

Supplementary Figure 3 | Charging profiles differentiated by model type for scenario 4 [S4: EV rate=80%]. Time-series analysis of daily charging loads for **(a)** PM-VF, **(b)** CCM, and **(c)** CEM. Quantitative assessment of relative change in output (VoSC, [%Δ]) for each model type **(d)** PM-VF, **(e)** CCM, and **(f)** CEM, measured against UCC, for the three key metrics max. peak demand [kWh], total charging costs [£], and total carbon emissions from charging [$kgCO_2$].

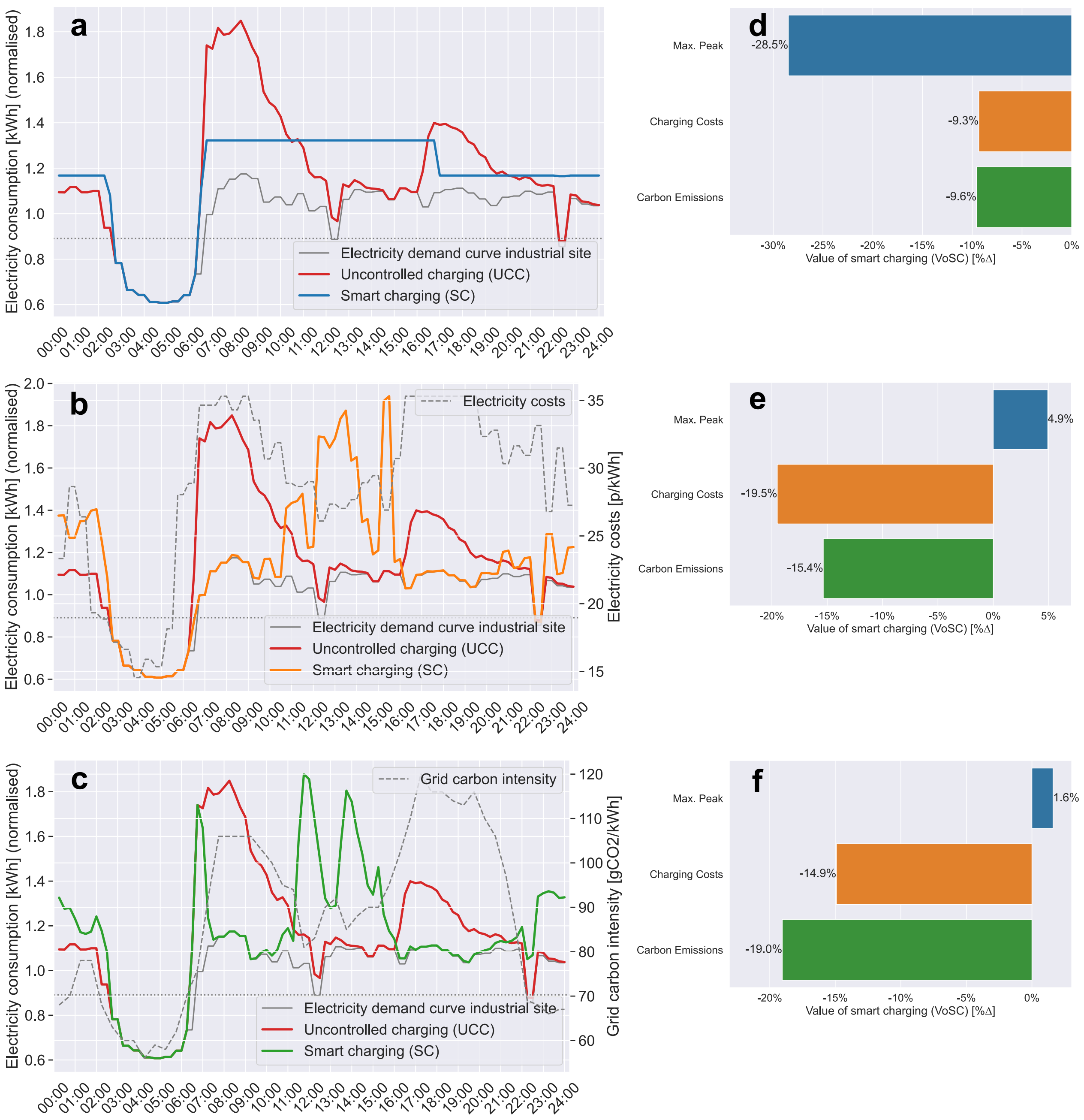

Supplementary Figure 4 | **Charging profiles differentiated by model type for scenario 5 [S5: EV rate=100%].** Time-series analysis of daily charging loads for **(a)** PM-VF, **(b)** CCM, and **(c)** CEM. Quantitative assessment of relative change in output (VoSC, [%Δ]) for each model type **(d)** PM-VF, **(e)** CCM, and **(f)** CEM, measured against UCC, for the three key metrics max. peak demand $[kWh]$, total charging costs [£], and total carbon emissions from charging $[kgCO_2]$.

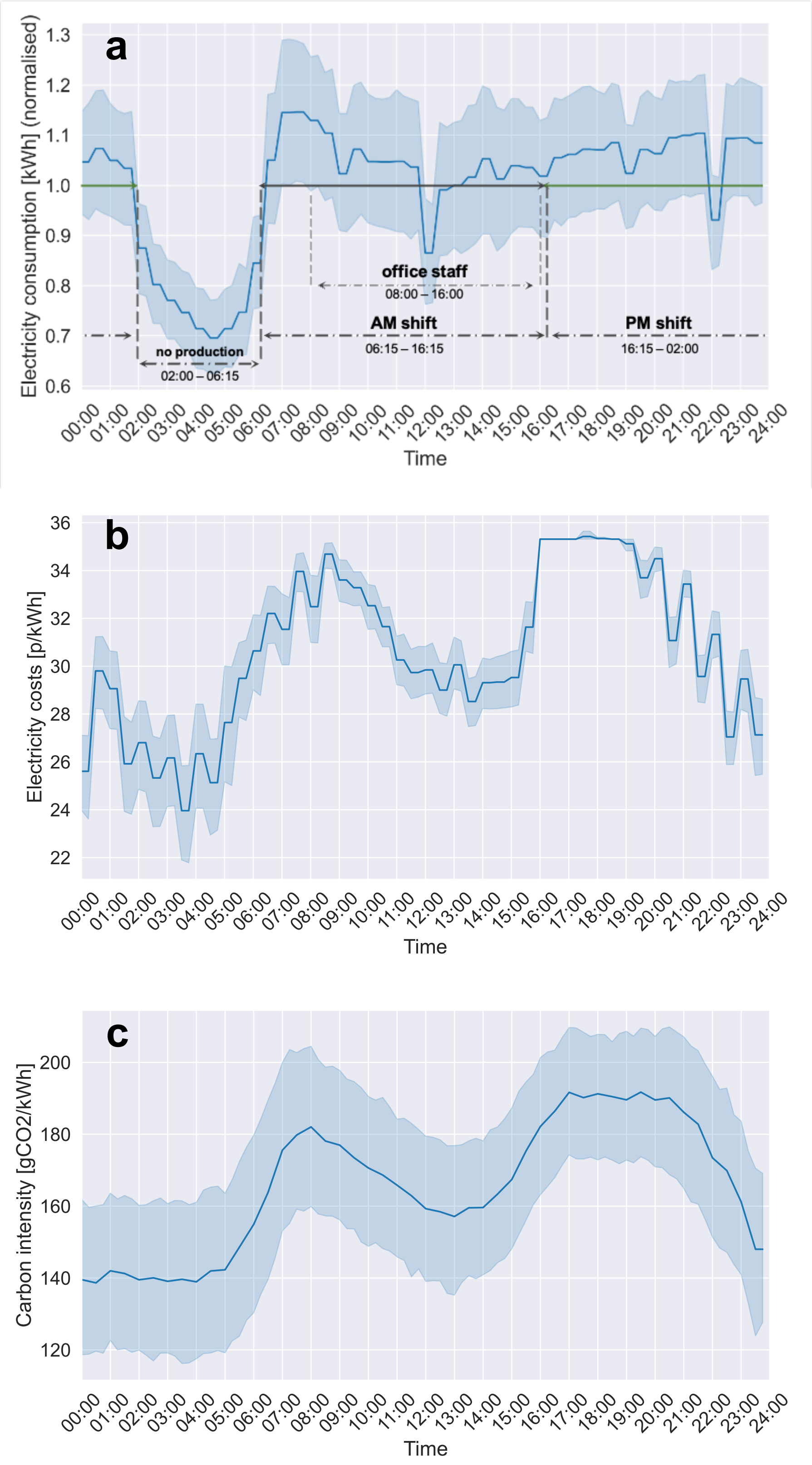

Supplementary Figure 5 | Time-series input data for February 2023. Summary of daily time-varying input parameters pertaining **(a)** firm-specific electricity consumption profile (P_t), **(b)** location-dependent Time-of-Use (ToU) tariff (λ_t), and **(c)** UK-wide grid carbon intensity (γ_t). Note: Uncertainty ranges indicate 95% confidence interval (CI).

Supplementary Table 1 | Numeric summary of key output metrics max. peak, charging costs and carbon emissions differentiated by model type for increasing EV rates [S1-5: 15–100%]. Quantitative assessment of output changes (VoSc, [%Δ]), measured against UCC, for PM-VF, CCM, CEM.

		Output changes (VoSC, [%Δ]) [1]				
Model type	Output metric	S1: 15%	S2: 30%	S3: 50%	S4: 80%	S5: 100%
PM-VF	max. peak	-7.4	-14.7	-21.3	-25.9	-28.5
	charging costs	-8.9	-10.1	-10.0	-9.6	-9.3
	carbon emissions	-10.5	-11.2	-10.7	-9.9	-9.6
CCM	max. peak	-3.2	-1.6	0.5	3.3	4.9
	charging costs	-18.5	-19.1	-19.6	-19.6	-19.5
	carbon emissions	-13.5	-14.8	-15.6	-15.3	-15.4
CEM	max. peak	-6.0	-5.3	-4.1	-0.5	1.6
	charging costs	-14.3	-14.7	-15.1	-14.9	-14.9
	carbon emissions	-17.4	-18.5	-19.3	-19.0	-19.0

[1]Note: In response to increasing EV adoption rates [S1-5: 15–100%].

Supplementary References

[1] Hart, W.E., Laird, C.D., Watson, J.-P., Woodruff, D.L., Hackebeil, G.A., Nicholson, B.L., Siirola, J.D.: Pyomo — Optimization Modeling in Python. Springer Optimization and Its Applications, vol. 67. Springer, Cham (2017). https://doi.org/10.1007/978-3-319-58821-6

[2] Streamlit: A faster way to build and share data apps (2024). https://streamlit.io/

[3] Octopus Energy: API Documentation (2024). https://developer.octopus.energy/

[4] NationalGridESO: Carbon Intensity API (2024). https://carbonintensity.org.uk/

[5] Gurobi Optimization, LLC: Gurobi Optimizer (2024). https://www.gurobi.com/
